# Physicochemical Properties and Consumer Acceptance of Bread Enriched with Alternative Proteins

**DOI:** 10.3390/foods9070933

**Published:** 2020-07-15

**Authors:** Purificación García-Segovia, Marta Igual, Javier Martínez-Monzó

**Affiliations:** Food Technology Department, Universitat Politècnica de València, Camino de Vera s/n, 46021 Valencia, Spain; marigra@upvnet.upv.es (M.I.); xmartine@tal.upv.es (J.M.-M.)

**Keywords:** alternative proteins, insect powder, pea protein, bread, Alveographic properties, physicochemical properties, sensory analysis, word association

## Abstract

A projected global population growth by 2050 and climate change crises have led to increasing demand in edible protein sources; thus, scientific research and food industries are searching for alternatives. In this study, we investigated the incorporation of plant- and insect-based protein sources in wheat-based formulations. The Alveographic properties of dough and the effects on bread physicochemical and sensory characteristics were analysed. Including pea protein or insect powder improved the nutritional value, increasing protein content, but influenced the dough and bread properties. Pea protein significantly increased the dough extensibility (L), tenacity (P), and their ratio (P/L) in dough with insect blends and the control. Bread texture properties were significantly affected by the addition of pea and insect flour. Higher amounts of pea protein incorporation increased hardness values and showed a mean cell area lower than the control bread. Crust colour analysis showed significant differences concerning the control bread, while crumb colour was affected by the flour colour. Word association analysis showed insect bread was associated with an emotional dimension, wheat bread was linked with “tradition”, and pea bread was associated with “fruit and vegetable”.

## 1. Introduction

The United Nations projected the global population will grow to between 8.4–8.7 billion in 2030, 9.4–10.2 billion in 2050, and reach 13.2 billion by 2100. In parallel, global life expectancy from birth is projected to rise to 77 years by 2045–2050 [1]. The challenges exposed by these projections also relate to the impact of large-scale environmental changes, and the need to maintain food supplies for an increasingly growing and expectant world population [2].

Because of this increasingly global and ageing population, there will be an increase in the demand for protein rich foods. Proteins are essential to maintain muscle mass and strength, especially in the elderly [3,4] but also to promote healthy growth in children [5], to obtain an optimal amount and maintain bone healthiness across all life stages [6], and to improve the adaptive response to training for athletes and fitness-minded individuals [7].

To balance climate change and the increasing protein demands, scientific research and food industries are investigating alternative protein sources (plant-based, insect-based, or cultured-meat) as ingredients for developing protein-rich foods [8,9,10]. A recent review discussed sustainable protein resources from marine, plant, dairy, and meat as well as novel sources including insects, rapeseed/canola, cereal, or cultured cells [11]. Besides, previous authors reported technologically functional, physicochemical, nutritional, and healthy properties of alternative protein sources in different food matrices [11,12,13,14,15,16,17,18,19,20] like in bakery products, pasta, yoghurt, snacks, burgers, and beverages.

Bread is one of the most consumed products worldwide, with its long history evolving through many forms, adopting different processes, different dough making formulas, and different ingredients [21]. Consumer interest in health and wellbeing is currently driving innovation in the bread sector; where it is already possible to find a varied choice of bread with added wholegrain, seeds, and high fibre. Partial substitution of wheat flour with alternative protein flours offers a viable method for increasing protein in the diet, particularly in countries with high consumption [22]; thus increases innovation in the bread sector. Several studies report the effects on value-added food products using alternative protein sources (plant- and insect-based) incorporated in bread, stating the improvement of physical, sensory, and nutraceutical characteristics [3,13,16,19,20,23,24,25,26,27,28,29,30,31].

A transition towards a plant-based diet has potential benefits for health and the environment [11,32,33]. Legumes and pulses are important in many diets because of their high protein content, low cost, and worldwide production. Peas (*Pisum sativum* L.) are produced primarily for human consumption; the major producers include Russia, China, Canada, Europe, Australia, and USA [34,35]. Peas contain 20–30% protein and are among the most widely cultivated and consumed pulse worldwide. Authors have studied pulse flour incorporation in food products, especially in bakery products [16,34,35,36]. These studies suggested that pulse flours were useful for designing new protein and fibre enriched food. Additionally, phenolic acids are the most important group of antioxidants presented in peas [37,38]. Furthermore, authors have reported evidence related to cholesterol impact [39], satiety and weight management [40], and muscle repair [41] showing pea protein’s potential health benefit. In the food industry, pea protein is used as a “source of protein” or “high protein” claim, particularly in dairy food products, when an increase in novel protein-rich foods, aimed at the sport-minded and elderly, are produced. In addition, bread fortification with pea protein allows the food industry to launch a “high protein” claim without increasing the gluten level, that effects dough texture, consistency, and elasticity while offering a complementary amino acid profile [42].

In January 2018, the European Union (EU) recognised insects as a novel food. Among the 12 insect species allowed in the EU are the mealworm (*Tenebrio molitor*) and buffalo worm (*Alphitobius diaperinus*) [43]. Insects are a food for the future and are already part of the diet for 2.5 billion people worldwide. The FAO also considers insects as healthy, nutritious, efficient, and sustainable foods [8,43]. Edible insects are a considerable source of protein and also contribute to fat, minerals, and vitamins; especially the B group; and fibre (especially chitin) intake [43]. Therefore, insects provide a promising novel resource for the food chain, including applications in human food. Authors have also investigated the inclusion of different insects in food matrices to show their nutritional benefits. Caparros Megido el al. [44] added whole mealworm larvae to burgers, whereas Choi et al. [45] used dried yellow mealworm larvae to replace pork meat in frankfurters; furthermore, authors also varied insect processing in tortilla chips [46,47]. Moreover, authors characterised insect protein extracts [48,49] or fats, and Delicato et al. [50] explored the consumer’s perception of bakery product by partially replacing butter with insect fat extract.

The development of sustainable food products offers a solution to many challenges in the food industry, including consumer acceptance. Often, in the food industry, new product development and academic research, sensory characterisation is used to understand the processes underlying consumer perceptions; with descriptive analysis techniques, applied with trained assessors, being most common [51]. However, consumer preference, attitudes, and food choice are subjective [52]. Several new sensory characterisation methodologies with consumers have been developed and reported, as reliable quick options, to understand consumer perception of new food products [51]; one such method is word association (WA). Roininen et al. [53] first applied this method to food concepts, and more studies have evaluated the applicability of WA for the evaluation of consumer’s perception of food products [54,55,56,57,58,59,60,61,62,63,64,65,66]. WA is based in freely elicited associations from consumers, in response to a stimulus. For food products, first associations might be the most relevant for consumers and hence drive their product choice and purchase decisions [53]. However, the most frequently elicited concepts may be the strongest and most important in the consumer’s mind [59].

Other reported studies have related sensory properties in bread made with alternative protein sources, and many have been evaluated by trained or untrained assessors [19,20,24,31,67,68,69,70,71,72]; also, the authors evaluated overall acceptance [29,73,74] with untrained assessors. Castro and Chambers [75] presented results of specific consumer behaviours toward insect-based products and Roma et al. [76] provided a useful contribution to understanding how consumer’s features may affect different behaviours towards entomophagy. Several studies identified factors to explain the low insect consumption acceptance in Belgium [52], Switzerland [46], Hungary [77], Poland [78], and Germany [79]. Nevertheless, few studies about consumer perception in bread with alternative protein sources have been found [46,50]; with none presented in the Spanish population.

This study aims to investigate the effects on bread, with a partial substitution of alternative plant- and insect-based protein, toward developing an innovative product. Experimental bread was produced using blends of wheat flour with mealworm (*Tenebrio molitor*), buffalo (*Alphitobius diaperinus*) powder, and isolate pea protein (5 and 10%) to evaluate the bread’s physicochemical properties, and to investigate the perception of naïve consumers using a WA test and liking study.

## 2. Materials and Methods

### 2.1. Insect Powder, Pea Protein, and Wheat Flour

Bread wheat flour (Grupo Gallo, El Carpio, Córdoba, Spain), salt, and compressed fresh yeast were acquired in a local market. Mealworm (*Tenebrio molitor* L.) and buffalo worm (*Alphitobius diaperinus*) powder were purchased from Kreca Ento-Food BV (Ermelo, The Netherlands). Roquette Frères S.A (Beinifaio, València, Spain) provided pea protein (NUTRALYS^®^ S85F). Ascorbic acid (E-300; food grade) (Panreac, Spain) was also used in formulations.

### 2.2. Samples Preparation

Seven flour blends were prepared in sets of 300 g. Bread made from commercial wheat flour was the control (CWF). Two amounts of alternative protein (two insect powder and one pea protein) sources at 5 and 10% (*w*/*w* regardless of wheat flour content) were used to replace wheat flour. Samples are coded as P5F: Pea 5%; P10F: Pea 10%; TM5F: *T. molitor* 5%; TM10F: *T. molitor* 10%; AD5F: *A. diaperinus* 5%; and AD10F: *A. diaperinus* 10%. Blends were packed in sachets, to protect from humidity and were stored in darkness at 25 °C until use.

### 2.3. Rheological Properties of Dough

An Alveograph Chopin (Villeneuve La Garenne, France) was used to perform Alveographic measurements following the standard American Association of Cereal Chemists (AACC) method 54–30A [80]. The Alveograph parameters included the deformation energy (W), dough extensibility (L), tenacity (P), the index of swelling (G), and curve configuration ratio (P/L). Alveograph experiments were conducted in duplicate. Moisture (g water/100 g sample) was determined by vacuum oven drying at 70 °C until achieving a constant sample weight [81].

### 2.4. Bread Making Process

The bread formula comprised 300 g of flour or a blend, compressed yeast (5% of flour), salt (1.5% of flour), ascorbic acid (0.01% of flour), and 180 g of warm water at 30 °C [27,35]. All ingredients were mixed for 4.0 min at low speed (speed 2) in a Kenwood Chef (Kenwood Limited, New Lane, Havant, UK) using a dough hook accessory to ensure proper hydration of flour. The dough was rested for 10 min then was shaped into loaves (80 g), kneaded, and left to ferment (15 min) at 28 °C in 85% relative humidity in a fermentation chamber (Convotherm OES 6.06 mini CC, Convotherm Elektrogeräte GMBH, Eglfing, Germany). The dough was baked at 210 °C for 25 min in a steamer oven (Convotherm OES 6.06 mini CC, Convotherm Elektrogeräte GMBH, Eglfing, Germany) [26]. The bread was left to cool at room temperature until core temperature reached 25 °C before slicing.

Six samples of bread for each formulation were obtained. The process was replicated at least in triplicate to obtain enough samples to analyse the different parameters in this study.

### 2.5. Bread Characterisation and Technological Properties

Crude protein content (CP) was evaluated according to official method 990.03 of Association of Official Analytical Chemists International (AOAC) [81], Dumas method in a Leco CN628 Elemental Analyser (Leco Corporation, St. Joseph, MI, USA). Total ash content was determined following AOAC 920.153 procedures [81]. A sample of 500 mg was incinerated in an oven (Muffle P Selecta Mod.367PE) for 24 h at 550 °C, and the ash was gravimetrically quantified. A section from the internal central crumb was crumbled and the water activity was measured using an AquaLab Dewpoint Water Activity Meter 4TE (Decagon Devices, Inc., Pullman, USA) [81]. All the chemical analyses were performed, at least in triplicate.

Texture profile analysis (TPA) was performed in bread slices (25 mm width) using a TA-XTPlus Texture Analyser (Stable Micro Systems Ltd., Godalming, UK) and the Texture Exponent Lite 32 (version 4.0.8.0) software (Godalming, UK) was used to process data to give textural parameters. A cylindrical aluminium probe (SMS P/75, 7.5 cm in diameter) and a 50 kg load cell were used. The parameters of the assay were defined as crosshead speed 1.7 mm/s and 40% deformation of the original length [82]. Six textural parameters were determined from each curve: Hardness, springiness, cohesiveness, and chewiness [83]. Six different slices for each bread formulation were measured.

Bread crumb structure was measured by digital image analysis. Bread slices (10 mm width) were cut vertically and scanned at 300 dpi with an Epson SX420 (Seiko Epson Corporation, Suwa, Japan). The scanned image was analysed using the software Image J (http://rsb.info.nih.gov/ij/) [84]; using the contrast between the two phases (pores and solid part) in the image. Six samples were analysed for each formulation. The crumb grain features were chosen the number of cells in a square 20 × 20 mm, cell area (mm^2^), mean cell area (mm^2^), cell area/total area (%), and cells/cm^2^.

The tristimulus colour parameters L * (lightness), a * (redness to greenness), b * (yellowness to blueness), and ∆E of the baked loaves (crumb and crust) were determined using a digital colourimeter (Chroma Meter CR-400, Konica Minolta, Japan). The instrument settings were illuminant C and observer angle 10°. Each sample was measured six times on three bread slices (20 mm width) to minimise the heterogeneity produced by the ingredients.

For bread samples, the browning index (BI) in crust and crumb was calculated following Equation (1):

(1) $$BI=\left[\frac{\left(100\left(X-0.31\right)\right)}{0.172}\right]$$

where x is given by Equation (2):

(2) $$x=\frac{\left(a^{*}+1.75L^{*}\right)}{\left(5.654L^{*}+a^{*}-3.012b^{*}\right)}.$$

The BI represents the purity of brown colour and is reported as an important parameter in processes where enzymatic or non-enzymatic browning takes place during the baking process [85].

### 2.6. Consumer Test

#### 2.6.1. Word Association Task

A convenience sampling was used during word association (WA) preliminary research. It enables an inexpensive approximation to a specific topic giving a gross estimate of the results [86]. This non-probability method, often used in consumers science, provides valuable insights for new food developments [59].

Participants were recruited using a proprietary consumer database and/or through different systems, including social media. Two criteria were defined for selecting the participants: Not to be celiac and to be a bread consumer (at least once a week). The respondents accepted participation by signing a consent form, provided before starting the survey.

**Figure 1:**
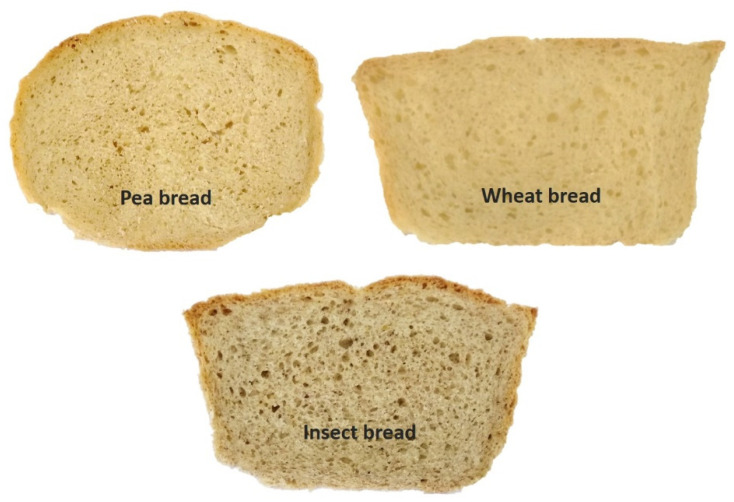
Stimulus presented to the respondent in an online questionnaire.

An online questionnaire was developed and sent by e-mail with RedJade^®^ (version v1.0.0.2) software (RedJade Sensory Solutions LLC, Martínez, CA, USA). The questionnaire was divided into two parts: The first comprised personal and socio-economic questions. The second included pictures of bread presented as stimuli (Figure 1). Pictures were used to assure that all participants faced the same stimulus for each bread [87]. Instructions given to participants (*n* = 327) were “Please write the four words, descriptions, associations, thoughts, and feelings that come to your mind when you see these pictures”.

#### 2.6.2. Liking Study

A liking study was conducted recruiting 106 consumers by e-mail from a proprietary consumer database. The inclusion criteria to participate were aged 20–50, in good health, available, presented no specific diet (such as medical or vegetarian), and consumed bread (at least ones a week). All participants signed an informed consent form and were rewarded for the time.

Standard sensory booths under controlled white light were used for the test (ISO 8589:2007). The seven samples of bread (CWB, P5B, P10B, TM5B, TM10B, AD5B, and AD10B) were codified with a three-digit number and were presented in a monadic sequence randomised with each participant. Participants were recommended to consume at least half of each bread loaf provided, before indicating their liking scores. A glass of water was provided to clean the palate between samples. The descriptors to evaluate were selected in accordance with previous sensory evaluation studies on bread and baked products [3,24,67,88]. Participants were asked to provide their liking responses for hardness (texture in the mouth), visual appearance, aroma (bread odour), taste, touch (consistency of the crumb to the touch) and overall liking. Liking responses were collected using a 9-point Likert scale from 1 “Dislike extremely” to 9 “Like extremely”. A quick questionnaire with demographic items including age, gender, and consumption frequency was included in the test.

### 2.7. Statistical Analysis

Two-way ANOVA and post-hoc Fisher’s least significant differences (LSD) were applied to establish significant statistical differences between samples. A principal component analysis (PCA) was conducted to identify relationships between dough characteristics, compositional parameters, and mechanical properties. To visualise the correlations between instrumental and hedonic attributes in the bread, a multi factor analysis (MFA) was conducted [89].

All respondents’ elicited words from the WA task were analysed qualitatively. Elicited words were grouped in distinct categories and the categories in dimensions. The grouping processes were performed by triangulation [90,91]. Frequencies of mention for each category were determined by counting the number of consumers that elicited similar words included in each category. Only those categories mentioned by over 10% of the respondents were considered. Differences in consumer perception according to bread type were evaluated using a chi-square test. Additionally, simple correspondence analysis was performed to better visualise the relationship between the stimuli and the elicited concepts.

All statistical analyses were performed with the XLSTAT 2020.1.2 software [89], and differences were considered significant at *p* < 0.05.

## 3. Results and Discussion

### 3.1. Rheological Properties of Dough

Table 1 shows the results (mean and standard deviation) of tenacity, extensibility, deformation energy, and the ratio of gluten behaviour obtained from the Alveograph. These parameters can help study the influence of the pea protein and insect powder addition on dough rheological properties [27,92,93].

Rheological properties of the blended flour bread were affected by the addition of the alternative protein [19,22,37,38,39,40]. The gluten network formation depends on gliadins and glutenin [21]. Glutenin is related to elasticity in dough development while gliadins contribute to the viscosity [94,95]. The extensibility (L) can be related to dough’s capacity to break down. Regarding L and G (Table 1) only the individual effect of flour type was statistically significant. Blends with insect powder, independent of species or quantity added, did not modify extensibility and the index of swelling, while the addition of pea protein significantly decreased (*p* < 0.05) the L and G of blended bread than the CWF. The addition of pea protein produced a negative effect on gliadin fraction reducing extensibility. This effect has been observed in similar studies with high protein flours [27,96], where the reduction in starch and gluten content was consistent with a reduction in L and G [97]. Tenacity (p) indicates the ability of the dough to retain gas [92]; here, Table 1 shows P varies significantly according to the flour type, percentage of substitution, and the two factors interaction. An increase in P was observed with the addition of pea protein (samples P5F and P10F) verifying a positive effect on the glutenin action [27]. A significantly lower value of P (*p* < 0.05) was found in the TM5F blend; however, in the TM10F and AD5F blends no significant differences (*p* > 0.05) relating to the CWF were found. The ratio between tenacity and extensibility (P/L) was used as an index of gluten behaviour or performance; an ideal P/L ratio for baking must be higher than 0.5 [93,96,98]. In the P/L ratio, individual effects and their interaction were statistically significant. The addition of pea protein significantly increased (*p* < 0.05) the P/L ratio over the insect powder blends and CWF. Regarding deformation energy (W), significant differences (*p* > 0.05) were not found for percentage of substitution, while the flour type and flour–percentage of substitution interaction was statistically significant (*p* < 0.05). According to Cappelli et al. [97] when blends with two insect species substituted with 5% and 10% were compared, a significant difference was not found. Nevertheless, a significant increase (*p* < 0.05) with blend P10F was observed. The addition of pea protein could contribute to forming a strong and rigid gluten network. The relatively low viscosity of pea protein can explain this effect. In contrast, insect flours do not contain starch and, despite their high protein content, influence the gluten network formation by lowering their strength [23,29,73,93].

### 3.2. Bread Characterisation and Technological Properties

Table 2 shows physicochemical and compositional parameters (a_w_, CP, and ash) of the experimental bread produced with CWF or blends containing pea protein and insect powder (P5B, P10B, TM5B, TM10B, AD5B, and AD10B). As expected, the addition of alternative protein sources affected the composition of bread. The individual effect of bread type and percentage of substitution in bread was significant for a_w_ and CP. The addition of high amounts of both insect powder and pea protein increases a_w_ and CP content, compared with the control bread (Table 2). Water activity is related to bread quality because it is highly related to the firming process in starch-based products [19]. The protein content of the alternative sources was 54.1% for *T. molitor*, 59.6% for *A. diaperinus*, and 84% for pea protein (commercial provider data). The higher increase in CP was observed in bread enriched with pea protein, while an increase in ash content was observed when 10% of insect powder was added to the dough. The addition of 5% of alternative protein did not show significant differences (*p* > 0.05) with alternative protein showed a higher protein and ash content than standard bread. in ash content. Besides, bread made with TM5B and TM10B showed higher values of a_w_. These results were in accordance with other authors, using insect flours like cricket (*Acheta domesticus*) [23,29], cinereous cockroach (*Nauphoeta cinerea*) [28], mealworm (*T. molitor* L.) [23,73], or plant-protein [35,39,40,47,48,49], bread enriched with pea protein.

Table 3a,b show technological properties (textural parameters, colour, and structural properties) of bread. TPA parameters revealed that the textural properties of bread were significantly affected (*p* < 0.05) by the addition of pea protein and insect powder. Regarding hardness, individual effects of bread type and percentage of substitution were observed. The interaction factors were also statistically significant (*p* < 0.05) (Table 3a) Insect powder addition did not significantly affect (*p* > 0.05) crumb hardness versus the control bread (AD10B ≤ AD5B = TM10B = TM5B ≤ CWB). Similar results were obtained by Gonzalez et al. [23] when wheat flour was replaced by cricket (*A. domesticus*) or mealworm (*T. molitor*) powder. Breads with pea protein increased crumb hardness in the order CWB < P5B < P10B (*p* < 0.05). These results were following other studies using plant-based proteins to obtain gluten-free bread [20,31,99] or fortify wheat bread with gums [27,100]. The effect of the two factors or their interaction was not significant (*p* > 0.05) for cohesiveness and springiness. Chewiness increased significantly (*p* < 0.05) with increased alternative protein incorporation, as did the significance of factors studied (Table 3a). This increasing in chewiness reveals that a long time is required for mastication before swallowing. Similar results were found by Ziobro et al. [20] using pea protein in gluten-free bread. Besides, lower chewiness values were obtained for insect bread and likewise, Gonzalez et al. [23] found no significant differences in chewiness comparing insect bread with wheat bread. These results were confirmed by the liking study; specifically, hardness evaluated by consumers showed a similar classification by score.

Bread crumb and crust colour were evaluated (Table 3b). Individual effects in bread type and percentage of substitution were statistically significant. Analysis of the crust colour showed significant differences (*p* < 0.05) in all CIELab parameters. *L* * values of the bread crust prepared with alternative proteins at 10% were significantly lower (*p* < 0.05) showing a darker bread. Whereas, a reduction in *L* * suggested an increase in Maillard-browning reactions because of, at least partly, high protein content [16]. For pea protein enriched bread, *a* * values were significantly higher (*p* < 0.05) resulting in redder bread, while bread TM5B and AD5B showed lower a * values than the control bread. Bread made with insect powder at high concentrations (10%) did not show significant differences (*p* > 0.05) in b * values compared to the control samples.

In contrast, the analysis of crumb colour showed no clear trends. L * crumb values of insect bread were significantly lower (*p* < 0.05) than the control bread and insect flour addition increase a * and b * values of crumb in *T. molitor* bread. These results agreed with previous studies where bread was produced with various insects species [23,28,73]. Pea protein bread shows a * and b * values significantly higher (*p* < 0.05) than the control, resulting in a loaf with a darker shade of yellow-red. Similar data were reported using pea protein in gluten-free bread [20,31] and baked crackers [16]. Thus, crumb colour is related to the flour colour, as the temperatures in the interior of the samples during the baking process did not reach 100 °C [23,100].

Here, ∆E > 3 implies a perceivable colour difference for a consumer [101]. As expected, crust and crumb in all breads exhibited ∆E > 3 than the control. Significantly higher (*p* < 0.05) ∆E values were obtained in the P10B crust. This result was agreed with other authors [15,16] which were attributed to the increase in Maillard-browning reactions with the increase in pea protein content. There were also significant differences in crumb colour, with ∆E > 3, and were therefore perceptible to the consumer.

**Figure 2:**
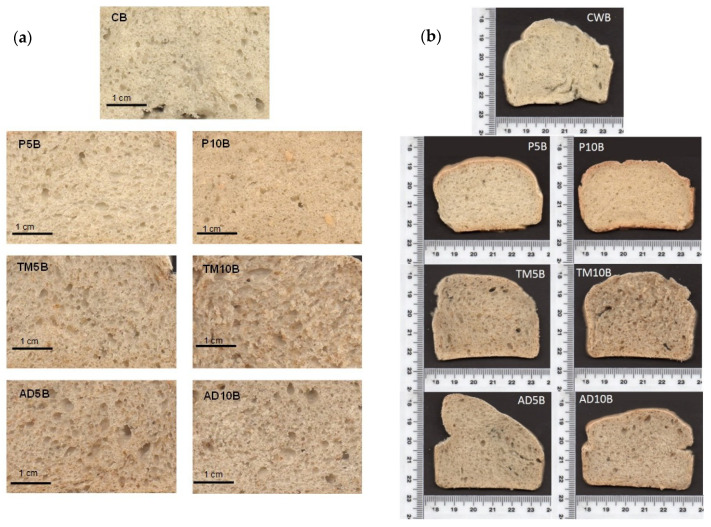
Digital analysis images of bread crumb (**a**) and cross-section (**b**) of bread made with alternative proteins. CWB: Control Bread; P5B: Pea 5% Bread; P10B: Pea 10% Bread; TM5B: *T. molitor* 5% Bread; TM10B: *T. molitor* 10% Bread; AD5B: *A. diaperinus* 5% Bread; AD10B: *A. diaperinus* 10% Bread.

Uniformity of the crumb was evaluated using digital image analysis. Table 3b shows the structural parameters, cell number, cell area, mean cell area, cell total area ratio, and number of cells/cm^2^. The effect of bread type was statistically significant, while not for the percentage of substitution. Pea protein bread showed a mean cell area lower than the control. For cell number (in × 400 mm^2^), significant differences (*p* < 0.05) were observed for most of the samples than the control (179 ± 15), with a lesser uneven distribution of gas cells found than in the control (Figure 2a). TM10B presented the highest cell area (145 mm^2^) and P5B the lowest (87 mm^2^). According to results in other insect breads [23], the addition of insect protein allows bread production with acceptable volume and a well-aired crumb. However, as can be seen in Figure 2b, air cells appear non-uniform and larger. This can be explained by the lower deformation energy (W) in insect protein doughs. This lower strength can contribute to the collapse of cells and the emergence of broken structures [102]. In contrast, pea protein impaired dough fermentation contributed to a more closed crumb and lower numbers of cells (Figure 2b) [31]. Likewise, a significant result was obtained for the bread-percentage of substitution interaction.

### 3.3. Consumer Test

#### 3.3.1. WA Task

The profile of the WA respondents is summarised in Table 4 and a majority (66.4%) consumed bread at least once a day. From the 327 respondents, 3924 terms, related to the three stimuli presented (Figure 1), were elicited with a mean of 3.7 words per stimulus and consumer. Terms were grouped into 586 valid codes, assembled into 112 categories, and these categories in 10 dimensions based on previous studies [54,66]. Only 23 categories, grouped in eight dimensions, were mentioned by at least 10% of the consumers [63], and were included in the analysis (Table 5). The chi-square test showed a significant correlation in the WA and the stimuli (χ^2^ = 1331.8; *p* < 0.0001). Besides, the application of the chi-square per cell was used to identify which categories were used for each type of stimulus contributing to the source of variation in the global chi-square [103].

WA is used in sensory science to understand how consumers perceive novel or abstract concepts [58]. This mental categorisation tool has been studied to develop new products [52,53,56,57,58,59,60,61], to evaluate new packaging [55,62], and undefined concepts [58,59,66,87]. These studies concluded that WA provided interesting and valuable information for gathering consumer perceptions [104].

In this study, the “No-sensory properties” dimension was more relevant for the three stimuli, while the “Original” category was the most repeated for pea and insect bread, and “Traditional” was the most mentioned category in wheat bread stimuli. In contrast, “Animals” (in the “Context” dimension) was the less repeated category for pea and wheat bread, while the “Specific food” dimension, especially “Fruit and vegetables”, was not associated with insect bread. Codes like “health”, “healthy”, and “beneficial for health” were significantly higher (*p* < 0.05), as expected, in pea bread stimuli, and significantly lower (*p* < 0.05) to the theoretical values for insect bread. Terms “Texture” and “Taste” were grouped in the “Sensory and hedonic” dimension and were significantly more (*p* < 0.05) elicited for wheat bread (Table 5). These results agreed with results of Gellynck et al. [105], who explored the quality perception in bread consumers. In this study, consumers perceive bread as a “basic” and “traditional” food, important in a “balanced diet” or as an “energy source” with a nutritional aspect, and “crispiness” and “taste” associated with sensory properties. Pontual et al. [63], assessing consumer expectations about pizza, obtained similar classifications as “new”, “sensory”, “specific food”, “health”, and “positive feelings”. From a cross-cultural perspective Sulmont-Rossé al. [66], investigating the concept of “feeling good” in a food context, found that the “sensory and hedonic” aspects were the most salient associations.

**Figure 3:**
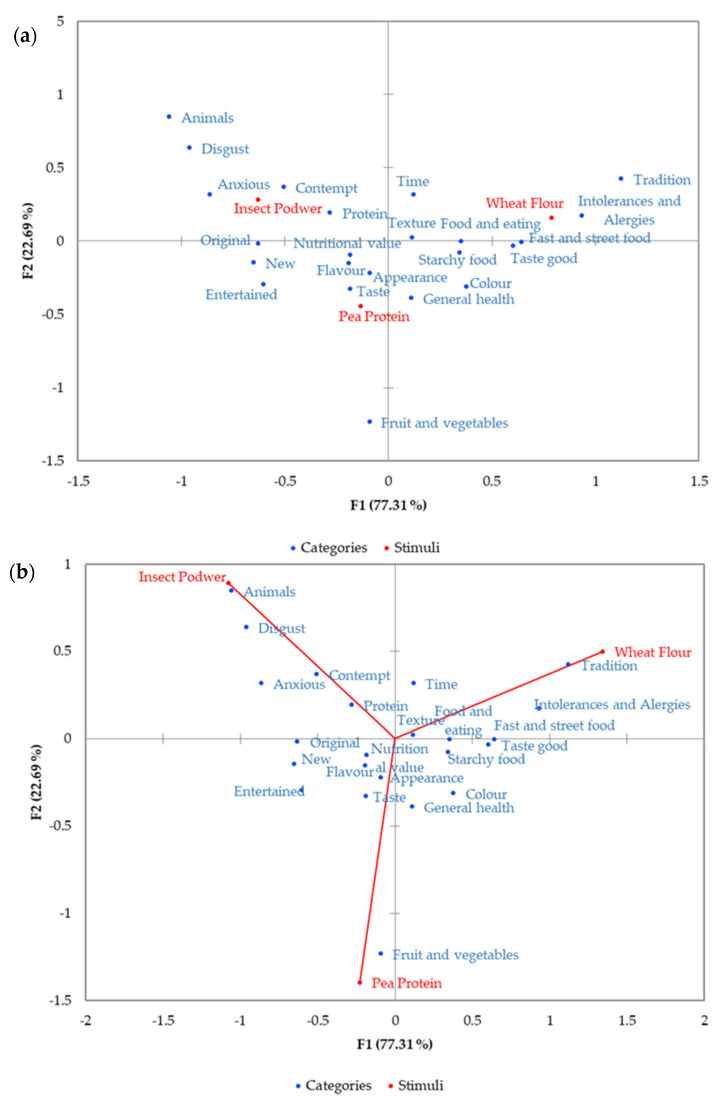
Correspondence analysis of contingency table. (**a**) Symmetric plot; (**b**) Asymmetric plot.

Figure 3 shows the correspondence analysis results according to mentioned relationships between types of bread and elicited associations represented in the same geometric space. In the symmetric plot (Figure 3a), bread was separated along the first axis. Insect bread was on the left, pea bread in the middle, and wheat bread on the right. Insect bread was associated with emotional dimensions (“contempt”, “disgust”, and “anxious” categories) and “animal”, correlated negatively with Factor 1 (F1). In contrast, wheat bread was linked with “tradition”, “intolerances and allergies”, “fast and street food”, and “taste good”, that positively contribute to F1. Factor 2 (F2), separated pea bread because of association with “fruit and vegetable” categories, as can be seen by their location in Figure 3b (asymmetric plot). 

#### 3.3.2. Liking Study

The liking study included 106 naïve consumers with at least 57.1% of them, frequent consumers of bread (criteria = once or more per day), and 14% consuming at least once a week.

Results of the ANOVA showing influence of the bread types on consumer preferences are presented in Table 6. Two breads made with 10% of insect powder obtained higher visual appearance scores, while a significant decrease (*p* < 0.05) in scores was observed in the order TM5B > AD5B > P10B > P5B > CWB. Taste of all insect bread was evaluated with significantly higher (*p* < 0.05) scores than the control and pea breads. Breads, TM5B and TM10B, were the most appreciated for the aroma attribute. Bread with pea protein (P10B and P5B) obtained a significantly higher score (*p* < 0.05) in hardness than insect bread. Similar results were obtained by Ziobro et al. [20,31], with non-gluten proteins in gluten-free bread, showing that introducing pea proteins caused a significant increase in acceptability of the bread smell, appearance, and taste.

Regarding overall acceptance, TM10B was the best evaluated with a score higher than five. In similar bread products, the overall liking score obtained for cricket bread was lower than *T. molitor* and *A. diaperinus* [29]; however, Roncolini et al. [73], showed better scores for *T. molitor*.

The influence of age and gender on the sensory parameters was also investigated, but no interaction was observed between the two factors.

### 3.4. Correlation Analysis

**Figure 4:**
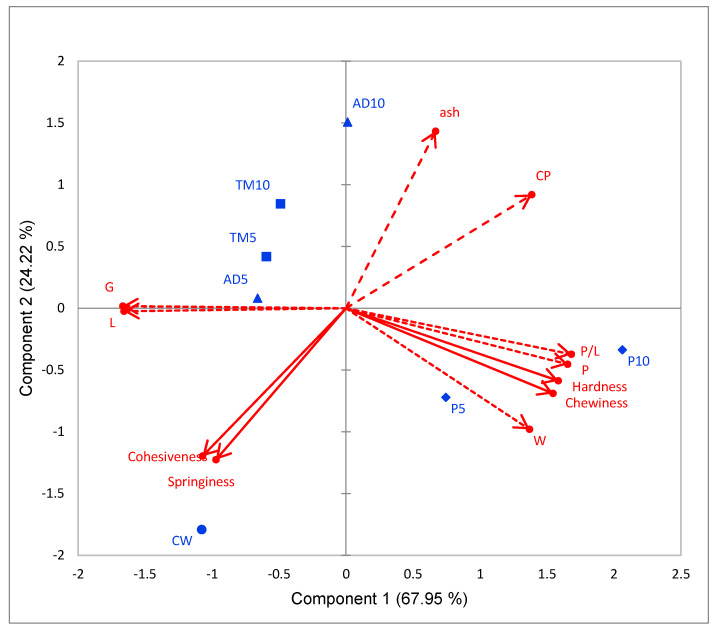
Biplot of principal component analysis (PCA) scores of samples and loadings of variables (rheological and compositional). CW: Control; P5: Pea 5%; P10: Pea 10%; TM5: *T. molitor* 5%; TM10: *T. molitor* 10%; AD5: *A. diaperinus* 5%; AD10: *A. diaperinus* 10%.

Principal component analysis (PCA) was conducted to identify relationships between dough properties, bread loaves characteristics, and compositional parameters (Figure 4). Globally, PC1 and PC2 explained 92.2% of the total variance. Compositional characteristics, hardness, chewiness, and the dough rheological properties (P, W, and P/L ratio) had positive loadings on PC1, whereas cohesiveness, springiness, G, and L had negative loading. PC2 was only negatively affected by the bread’s mechanical properties (cohesiveness and springiness). Crude Protein (CP) showed a significantly negative correlation with cohesiveness and springiness (r = −0.83 and r = −0.78, *p* < 0.05, respectively). P is positively affected by CP content (r = 0.63), that is negatively affected by dough extensibility. As expected, hardness showed a positive correlation with dough tenacity (r = 0.95) and deformation energy (r = 0.092), and was negatively correlated with dough extensibility (r = −0.83) and index of swelling (r = −0.85).

Based on PC1 and PC 2, the samples scores were distributed according to the type of protein. PC2 differentiated insect protein blends, while PC1 separated pea protein blends. Cohesiveness and springiness higher correlated with lower protein content, marked CW sample to lower left quarter on the plane by PC1 and PC2.

**Figure 5:**
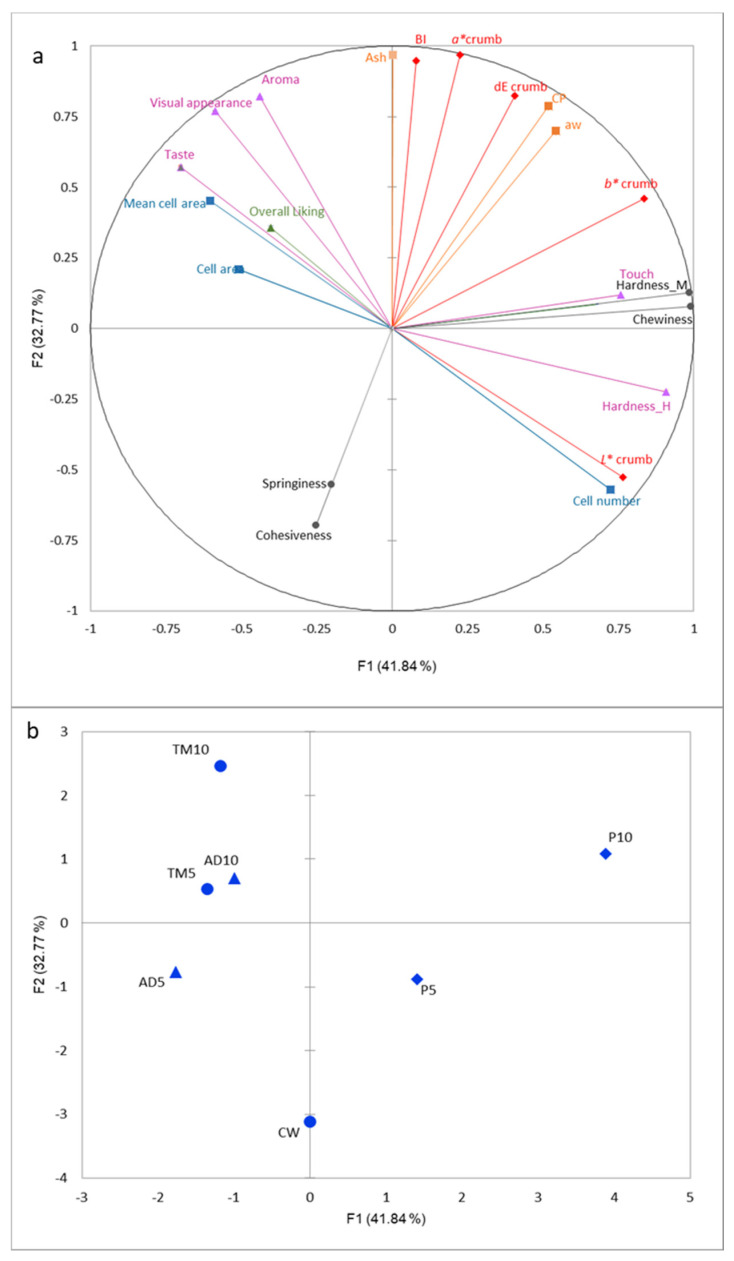
Multi factor analysis (MFA) score plot of loading plot of instrumental and hedonic attributes (**a**) and bread samples (**b**). CW: Control; P5: Pea 5%; P10: Pea 10%; TM5: *T. molitor* 5%; TM10: *T. molitor* 10%; AD5: *A. diaperinus* 5%; AD10: *A. diaperinus* 10%.

To identify the correlation between instrumental and hedonic results, an MFA was conducted (Figure 5). Mechanical and hedonic hardness were highly correlated (r = 0.90) as were hardness and touch (r = 0.72). Visual appearance was correlated with crumb cell (gas bubbles) and negatively correlated with luminosity of the crumb (r = −0.77). A higher negative correlation was observed between hedonic and instrumental hardness, cell area, mean cells area. RV coefficients showed the closest tables, comprised instrumental texture (RV = 0.80), colour crumb (RV = 0.794), and hedonic attributes (RV = 0.78). Globally, overall liking was positively associated with porosity (represented by cell area and mean cell) and negatively related with hardness and L* of crumb.

Distribution of samples in the bottom left in MFA plot comprises formulations containing insect protein which exhibit better hedonic scores, including an overall liking than the pea protein bread. In contrast, the pea bread was related to instrumental and hedonic hardness, and the control bread was separated from the other groups negatively affected by protein content.

## 4. Conclusions

Despite limitations, results of this study provide valuable information to produce enriched bread, especially regarding the few studies about buffalo powder (*Alphitobius diaperinus*) use as an ingredient. We showed the investigated alternative protein sources could be incorporated into bread to improve their protein content. In addition, the increased ash content coupled with the increase in percentage of substitution shows a possible improvement in mineral content. Following past literature, partial substitution of wheat flour with alternative proteins decreased bread quality. Although most technological functional properties did not differ between the two insect powders used, results in this study show improved values for *Tenebrio molitor* than previous studies.

Results from the WA task and liking scores gave interesting information about the acceptance of insect and pea enriched bread. Enriched bread with insect protein obtained higher liking score in attributes correlated with overall acceptance (Figure 5). However, to better investigate consumer perception and acceptability, more studies have been planned, testing consumption of alternative protein products in real eating environments

## Figures and Tables

**Table 1:** Alveographic characterisation of the control wheat flour (CWF) and six blends of wheat flour and alternative protein sources used for bread making.

	P (mmH_2_O)	L (mm)	G	W (10^−4^ J)	P/L
**CWF**	112 (4) ^de^	44 (2) ^a^	14.7 (0.3) ^a^	201 (8) ^b^	2.56 (0.10) ^d^
**P5F**	193 (8) ^b^	26 (2) ^c^	11.4 (0.5) ^c^	215 (12) ^b^	7.34 (0.41) ^b^
**P10F**	245 (3) ^a^	24 (1) ^c^	10.9 (0.1) ^c^	276 (3) ^a^	10.20 (0.11) ^a^
**TM5F**	96 (2) ^f^	38 (1) ^ab^	13.7 (0.2) ^ab^	150 (6) ^c^	2.53 (0.10) ^d^
**TM10F**	104 (4) ^ef^	39 (7) ^ab^	13.8 (1.2) ^ab^	129 (25) ^c^	2.74 (0.52) ^d^
**AD5F**	118 (7) ^d^	39 (5) ^ab^	13.8 (0.8) ^ab^	156 (25) ^c^	3.09 (0.50) ^cd^
**AD10F**	126 (2) ^c^	35 (6) ^b^	13.1 (1.2) ^b^	158 (24) ^c^	3.66 (0.57) ^c^
**Factor**	Significance				
**Flour type**	<0.0001	<0.0001	<0.0001	<0.0001	<0.0001
**% Substitution**	<0.0001	−	−	−	<0.0001
**Flour × % Substitution interaction**	<0.0001	−	−	0.003	<0.0001

CWF: Control Wheat Flour; P5F: Pea 5% Blend; P10F: Pea 10% Blend; TM5F: *Tenebrio* *molitor* 5% Blend; TM10F: *T. molitor* 10% Blend; AD5F: *Alphitobius* *diaperinus* 5% Blend; AD10F: *A. diaperinus* 10% Blend. ^a–f^ The same letter within column indicates homogeneous groups established by two-way ANOVA (*p* < 0.05) where (−) indicates no significant differences.

**Table 2:** Mean values (and standard deviations) of water activity, crude protein, and ash content of bread.

	a_w_	CP (g/100 g)	Ash (g/100 g)
**CWB**	0.887 (0.008) ^de^	13.33 (0.07) ^f^	1.08 (0.07) ^f^
**P5B**	0.896 (0.015) ^cd^	16.08 (0.07) ^d^	1.55 (0.06) ^cd^
**P10B**	0.922 (0.004) ^a^	19.31 (0.08) ^a^	1.70 (0.03) ^bc^
**TM5B**	0.907 (0.006) ^bc^	15.35 (0.12) ^e^	1.58 (0.04) ^cd^
**TM10B**	0.913 (0.003) ^ab^	17.27 (0.02) ^c^	1.91 (0.08) ^a^
**AD5B**	0.877 (0.003) ^e^	15.27 (0.03) ^e^	1.50 (0.08) ^e^
**AD10B**	0.894 (0.004) ^d^	17.53 (0.06) ^b^	1.75 (0.09) ^b^
**Factor**	Significance		
**Bread**	<0.0001	<0.0001	−
**% Substitution**	0.025	<0.0001	<0.0001
**Bread × % substitution interaction**	−	<0.0001	−

CWB: Control Bread; P5B: Pea 5% Bread; P10B: Pea 10% Bread; TM5B: *T. molitor* 5% Bread; TM10B: *T. molitor* 10% Bread; AD5B: *A. diaperinus* 5% Bread; AD10B: *A. diaperinus* 10% Bread. ^a–f^ The same letter in superscript within column indicates homogeneous groups established by two-way ANOVA (*p* < 0.05) where (−) indicates no significant differences.

**Table 3:** (**a**) Effect of alternative protein addition on technological properties of bread: Crumb texture parameters. (**b**) Effect of alternative protein addition on technological properties of bread: colour and structural properties.

(**a**)											
		**Hardness (g)**		**Cohesiveness**			**Springiness**			**Chewiness (g)**	
**CWB**		1360 (237) ^c^		0.879 (0.008) ^a^			97.5 (1.4) ^a^			1166 (207) ^c^	
**P5B**		2093 (493) ^b^		0.845 (0.030) ^ab^			96.2 (1.6) ^b^			1700 (399) ^b^	
**P10B**		3442 (790) ^a^		0.801(0.017) ^c^			95.34 (1.02) ^bc^			2617 (543) ^a^	
**TM5B**		1108 (103) ^cd^		0.821 (0.112) ^bc^			96.3 (2.3) ^ab^			879 (161) ^d^	
**TM10B**		1216 (97) ^cd^		0.841 (0.015) ^abc^			96.5 (1.2) ^ab^			985 (68) ^cd^	
**AD5B**		927 (155) ^d^		0.837 (0.012) ^bc^			96.4 (1.9) ^ab^			747 (124) ^d^	
**AD10B**		1037 (131) ^cd^		0.806 (0.022) ^bc^			94.4 (1.5) ^c^			789 (100) ^d^	
Factors		Significance									
Bread		<0.0001		−			−			<0.0001	
% Substitution		<0.0001		−			−			<0.0001	
%Substitution * Bread interaction		<0.0001		−			−			<0.0001	
(**b**)											
		**CWB**	**P5B**	**P10B**	**TM5B**	**TM10B**	**AD5B**	**AD10B**	**Pr > F** **(Bread)**	**Pr > F** **(% Substitution)**	**Pr > F** **(% Substitution × Bread)**
**Crust**	*L* *	74.6 (2.4) ^a^	70.1 (1.9) ^b^	66.2 (2.8) ^c^	73.5 (2.5) ^a^	67.7 (3.9) ^bc^	73.9 (1.6) ^a^	67.7 (1.1) ^bc^	0.020	<0.0001	−
	*a* *	6.4 (2.1) ^cd^	9.7 (0.8) ^b^	12.2 (1.1) ^a^	5.2 (1.4) ^de^	6.4 (1.1) ^cd^	3.8 (1.2) ^e^	7.6 (0.9) ^c^	<0.0001	<0.0001	0.049
	*b* *	26.9 (3.6) ^bc^	30.9 (0.7) ^a^	32.3 (1.2) ^a^	24.7 (1.9) ^c^	26.2 (1.8) ^bc^	20.4 (2.3) ^d^	27.2 (1.3) ^b^	<0.0001	<0.0001	0.003
	ΔE	−	7.1 ^b^	11.6 ^a^	3.8 ^c^	7.4 ^b^	7.4 ^b^	7.1 ^b^	0.001	0.001	0.024
	BI	49.7 (11.2) ^cd^	65.9 (3.6) ^b^	77.5 (8.2) ^a^	44.7 (6.5) ^d^	54.4 (9.5) ^c^	34.6 (2.2) ^e^	57.3 (4.5) ^c^	<0.0001	<0.0001	0.070
**Crumb**	*L* *	70.7 (2.4) ^ab^	68.9 (1.7) ^bc^	72.7 (1.1) ^a^	65.1 (2.4) ^d^	62.2 (3.6) ^e^	67.2 (2.2) ^cd^	67.5 (2.7) ^cd^	<0.0001	−	0.007
	*a* *	1.2 (0.2) ^e^	2.56 (0.11) ^c^	3.5 (0.2) ^a^	2.8 (0.3) ^bc^	3.9 (0.6) ^a^	2.00 (0.13) ^d^	2.97 (0.14) ^b^	<0.0001	<0.0001	−
	*b* *	16.7 (0.5) ^d^	19.9 (0.8) ^b^	22.9 (0.5) ^a^	18.5 (0.6) ^c^	19.2 (1.7) ^c^	17.3 (0.6) ^d^	16.9 (0.6) ^d^	<0.0001	0.0002	<0.0001
	ΔE	−	4.2 ^cd^	6.9 ^b^	6.2 ^b^	9.4^a^	3.8 ^d^	3.9 ^cd^	<0.0001	0.003	−
	BI	27.1 (2.1) ^d^	35.2 (0.7) ^b^	39.7 (1.4) ^a^	35.1 (2.2) ^b^	40.2 (5.6) ^a^	30.61 (1.02) ^c^	30.8 (1.6) ^c^	<0.0001	0.001	−
**Crumb** **digital image**	Cell number *	179 (15) ^a^	155 (5) ^bc^	175 (5) ^ab^	137 (19) ^cde^	119 (29) ^e^	130 (19) ^de^	147 (7) ^cd^	<0.0001	−	0.016
	Cell area (mm^2^)	115 (20) ^bc^	87 (16) ^d^	97 (26) ^cd^	96 (16) ^cd^	145 (30) ^a^	128 (27) ^ab^	99 (8) ^cd^	0.012	−	<0.001
	Mean cell area (mm^2^)	0.66 (0.15) ^b^	0.56 (0.09) ^b^	0.56 (0.16) ^b^	0.71 (0.14) ^b^	1.30 (0.49) ^a^	1,01 (0.27) ^a^	0.68 (0.08) ^b^	0.001	−	<0.001
	Cell area/total area (%)	29 (5) ^bc^	22 (4) ^d^	24 (7) ^cd^	24 (4) ^cd^	36 (8) ^a^	32 (7) ^ab^	25 (2) ^cd^	0.012	−	<0.001
	Cells/cm^2^	44.7 (3.7) ^a^	38.8 (1.2) ^bc^	43.6 (1.3) ^ab^	34.3 (4.8) ^cde^	29.7 (7.2) ^e^	33.3 (4.7) ^de^	36.8 (1.9) ^cd^	<0.0001	−	0.016

CWB: Control Bread; P5B: Pea 5% Bread; P10B: Pea 10% Bread; TM5B: *T. molitor* 5% Bread; TM10B: *T. molitor* 10% Bread; AD5B: *A. diaperinus* 5% Bread; AD10B: *A. diaperinus* 10% Bread. * Measured in 20 × 20 mm square fields of view of the central slice. ^a–^^e^ The same letter in superscript within column indicates homogeneous groups established by two-way ANOVA (*p* < 0.05) where (−) indicates no significant differences.

**Table 4:** Characteristics of the respondent at word association questionnaire.

	Variable	
**Sex**	Female	66.4%
	Male	33.3%
	n/a	0.3%
Age *	Female	26 (11)
	Male	30 (13)
**Origin**	Valencian	50.2%
	Spanish	37.9%
	Latin American	9.8%
	European	2.1%
**Work status**	Student	55.4%
	Employed	39.1%
	Unemployed	2.1%
	Retired	0.6%
	Other	2.8%
Bread consumption **	Never	0.9%
	Several times a day	36.1%
	Once a day	30.3%
	Several times a week	21.6%
	Several times a month	5.6%
	Occasionally	5.6%

* Mean (SD), ** Measured on a 5-point scale with 0-several times a day. 1-once a day; 2-several times a week; 3-once a week; 4-several times a month; 5-occasionally.

**Table 5:** Contingency table with the main dimensions, categories and example of codes elicited by respondents to bread presented; the number of citations per stimuli and results of the Chi-squared per cell test.

Dimensions	Categories ^(1)^	Examples of Codes	Pea Bread ^(2)^	Insect Bread ^(2)^	Wheat Bread ^(2)^	Χ^2 (3)^
Body and health	General health	Health, healthy, beneficial for health	84(+)	30(−)	52	27.4
	Intolerances and Allergies	Allergens, gluten, celiac, intolerance	11(−)	3(−)	46(+)	54.2
Context	Time	Breakfast, lunch, dinner, never	13(−)	29	32	8.6
	Animals	Bee, insect, bug, butterfly, cockroach, chicken	1(−)	52(+)	0(−)	97.2
Cooking and eating	Food and eating	Food, meal, starter, appetiser, garnish	25	17(−)	39(+)	10.0
Feelings and emotions	Entertained	Funny, interesting	35(+)	32(+)	1(−)	30.5
	Anxious	Impatient, nervous, restless, inquiring	18	54(+)	0(−)	60.7
	Contempt	Indifference, disregarding, contempt	17(−)	54(+)	14(−)	33.2
	Disgust	Dislike, disgust, repugnance, aversion	12(−)	97(+)	1(−)	145.7
Non-sensory properties	Original	Original, different, strange, curious, rare	131(+)	190(+)	16(−)	133.6
	New	New, novel, innovative, creative	80(+)	95(+)	3(−)	79.4
	Tradition	Basic, classic, conventional, traditional	13(−)	15(−)	229(+)	370.7
Nutrition	Nutritional value	Nutrients, nutritional, energy, complete food	32	31	19(−)	3.5
	Protein	Protein, proteic, full protein	18	34(+)	16	7.8
Sensory and hedonic properties	Appearance	Good appearance, compact, attractive	107(+)	74	61(−)	13.7
	Texture	Soft, crunch, hard, gummy, fluffy	101	98	123(+)	4.5
	Taste good	Good, delicious, tasty, palatable, savoury	67	24(−)	130(+)	81.3
	Colour	Yellow, white, green, brown	67(+)	16(−)	66(+)	35.9
	Taste	Tasteless, taste, bland	39(+)	24	15(+)	11.1
	Flavour	Aromatic, stale, traditional flavour	23	20	12	3.3
Specific food	Fruit and vegetables	Garlic, peas, tomato, vegetables, fig	42(+)	0(−)	4(−)	70.3
	Starchy food	Flour, crust, crumb, bread, sandwich loaf	36	20(−)	49(+)	13.1
	Fast and street food	Sandwich, pizza, toast, hamburger	25	9(−)	53(+)	35.9
		χ^2^ ^(4)^	213.9	484.5	633.4	1331.8

^(1)^ Only categories mentioned by over 10% of the respondents at least in one stimulus were considered. ^(2)^ Symbol next to the actual value indicates if it is significantly lower (−) or higher (+) than the theoretical value according to chi-square tests (*p* < 0.05). ^(3) (4)^ Total Chi-square by rows and columns, respectively.

**Table 6:** Hedonic data of liking study (*n* = 106).

	Visual Appearance	Aroma	Taste	Touch	Hardness	Overall Liking
**CWB**	1.3 (0.6) ^f^	3.2 (2.1) ^d^	4.5 (1.8) ^bc^	5.3 (2.1) ^b^	5.2 (2.3) ^ab^	5.3 (0.9) ^b^
**P5B**	2.2 (0.9) ^e^	3.6 (2.2) ^d^	3.6 (1.8) ^d^	4.3 (2.4) ^c^	5.6 (2.1) ^a^	3.8 (1.3) ^e^
**P10B**	3.3 (1.2) ^d^	4.1 (2.5) ^d^	4.4 (1.8) ^c^	6.2 (2.2) ^a^	5.9 (2.2) ^a^	4.1 (1.6) ^de^
**TM5B**	6.1 (1.1) ^b^	6.1 (2.1) ^b^	5.5 (1.4) ^a^	4.2 (2.3) ^c^	4.2 (1.8) ^c^	5.1 (0.9) ^bc^
**TM10B**	8.1 (1.1) ^a^	7.3 (1.9) ^a^	5.8 (2.4) ^a^	4.9 (2.1) ^bc^	4.6 (1.9) ^bc^	6.6 (1.1) ^a^
**AD5B**	5.4 (0.9) ^c^	3.9 (2.1) ^d^	5.1 (1.8) ^ab^	4.1 (2.3) ^c^	4.4 (2.2) ^bc^	3.8 (1.2) ^e^
**AD10B**	7.9 (1.1) ^a^	5.2 (2.3) ^c^	5.8 (2.2) ^a^	4.5 (2.3) ^bc^	4.1 (1.9) ^c^	4.7 (1.5) ^cd^

CWB: Control Bread; P5B: Pea 5% Bread; P10B: Pea 10% Bread; TM5B: *T. molitor* 5% Bread; TM10B: *T. molit* or 10% Bread; AD5B: *A. diaperinus* 5% Bread; AD10B: *A. diaperinus* 10 % Bread. ^a–e^ The same letter in superscript within the column indicates no significant difference according to Tukey HSD test (*p* < 0.05).
